# An Investigation into the Association between DNA Damage and Dietary Fatty Acid in Men with Prostate Cancer

**DOI:** 10.3390/nu7010405

**Published:** 2015-01-08

**Authors:** Karen S. Bishop, Sharon Erdrich, Nishi Karunasinghe, Dug Yeo Han, Shuotun Zhu, Amalini Jesuthasan, Lynnette R. Ferguson

**Affiliations:** 1 Auckland Cancer Society Research Centre, FM & HS, University of Auckland, Private Bag 92019, Auckland 1142, New Zealand; E-Mails: n.karunasinghe@auckland.ac.nz (N.K.); l.ferguson@auckland.ac.nz (L.R.F.); 2 Discipline of Nutrition, FM & HS, University of Auckland, Private Bag 92019, Auckland 1142, New Zealand; E-Mail: sharon.erdrich@gmail.com; 3 Nutrigenomics New Zealand, University of Auckland, Private Bag 92019, Auckland 1142, New Zealand; E-Mails: dy.han@auckland.ac.nz (D.Y.H.); st.zhu@auckland.ac.nz (S.Z.); amalini3@hotmail.com (A.J.)

**Keywords:** DNA damage, Mediterranean style diet, fatty acids, prostate cancer

## Abstract

Prostate cancer is a growing problem in New Zealand and worldwide, as populations adopt a Western style dietary pattern. In particular, dietary fat is believed to be associated with oxidative stress, which in turn may be associated with cancer risk and development. In addition, DNA damage is associated with the risk of various cancers, and is regarded as an ideal biomarker for the assessment of the influence of foods on cancer. In the study presented here, 20 men with prostate cancer adhered to a modified Mediterranean style diet for three months. Dietary records, blood fatty acid levels, prostate specific antigen, C-reactive protein and DNA damage were assessed pre- and post-intervention. DNA damage was inversely correlated with dietary adherence (*p* = 0.013) and whole blood monounsaturated fatty acids (*p* = 0.009) and oleic acid (*p* = 0.020). DNA damage was positively correlated with the intake of dairy products (*p* = 0.043), red meat (*p* = 0.007) and whole blood omega-6 polyunsaturated fatty acids (*p* = 0.015). Both the source and type of dietary fat changed significantly over the course of the dietary intervention. Levels of DNA damage were correlated with various dietary fat sources and types of dietary fat.

## 1. Introduction

Prostate cancer in New Zealand and worldwide is an increasing problem with respect to prevalence and receipt of appropriate, and in some countries, timely treatment. Prostate cancer is the most common cancer amongst men in New Zealand, accounting for 27% of all new male cancer cases [1]. In addition to older age, ethnicity and family history being risk factors for prostate cancer, lifestyle is also believed to play a role [2]. This belief is supported by evidence obtained from migrants who adopted the lifestyle of their new country to varying degrees [3]. Such migrants also adopted the risk levels associated with that country, rather than their country of origin, depending on the extent to which they changed their lifestyle [3]. It is widely accepted that diet plays an important role in the development of cancers and that a Mediterranean style diet, as opposed to a Western style diet, may ameliorate the risk and progression of prostate cancer due to the effect of various Mediterranean style dietary components on inflammation and oxidative stress, amongst other factors [4]. The source and components of dietary fat vary enormously between Mediterranean and Western dietary patterns. The former is higher in monounsaturated fatty acid (MUFA) rich-plant foods including oleic acid-rich olive oils, as well as the long chain omega 3 polyunsaturated fatty acids (PUFA) that are largely sourced from oily fish (which are high in the omega 3 fatty acids (*n*3PUFA), eicosapentanoiec acid (EPA) and docosahexaneoic acid (DHA)) [5]. A Western style dietary pattern on the other hand is higher in omega 6 fatty acids (*n*6PUFA) sourced largely from seed oils and animal fats [5].

Exogenous and endogenous factors can influence oxidative stress [6], which is caused by an imbalance between antioxidants and reactive oxygen species. Lifestyle and diet can be a source of antioxidants and can also promote oxidative stress. Examples of foods that promote oxidative stress include meat cooked at high temperature, as well as some processed and smoked meats [7,8]. Meat cooked at high heat can generate heterocyclic amines (HCA) and polycyclic aromatic hydrocarbons and these can induce DNA instability [7,8,9,10]. The susceptibility to prostate cancer risk as a result of consumption of such compounds may be modified by genotype [11]. The consumption of processed meats may also promote the formation of cancers as they contain potentially harmful nitrates and nitrites [9]. Other dietary sources of fat, such as dairy, contain calcium and angiotensin-converting enzyme inhibitors that may decrease oxidative stress, at least in people who are obese [12]. Despite such evidence, dairy intake has received mixed reviews with respect to association with prostate cancer risk [13,14,15].

There is some controversy regarding dietary fat intake and prostate cancer prevalence and progression [16,17,18]. Total and saturated fat intake has been positively associated with prostate specific antigen (PSA) levels [19], increased risk of prostate cancer, and aggressive prostate cancer [16,18], whilst saturated fat intake has been associated with fatal prostate cancer [18].

**Figure 1:**
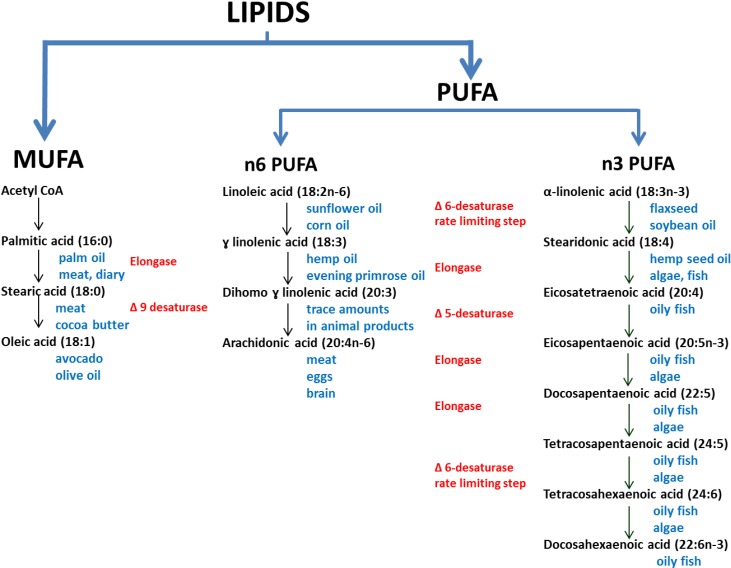
The biosynthesis pathways of the omega 3, 6 and 9 family of poly- and mono-unsaturated fatty acids (adapted from [21,22,23]).The main dietary sources are shown in blue text, and the enzymes in red text.

The dietary fatty acids that are discussed herein are shown in relation to one another in Figure 1. Both animal and plants consist of different types of fats in varying proportions. Animal fats consist predominantly of saturated fats (single carbon bonds in the hydrocarbon chains), and plant fats consist predominantly of unsaturated fats (with a varying number of double bonds). There are some exceptions, for example coconut oil contains predominantly saturated fat, and fish consists primarily of PUFA. Unsaturated trans fats are only found in trace amounts in meat and dairy, but they are often produced during the hydrogenation of vegetable oils to produce saturated fats, and therefore are common in processed foods [20].

Linoleic and α-linolenic acid are essential fatty acids, whilst other fatty acids, to some degree, can be synthesised from precursors [22] (Figure 1). In the *n*3 PUFA and *n*6PUFA pathways there is competition for the ∆6-desaturase and ∆5-desaturase enzymes, although both enzymes preferentially catalyse the *n*3PUFA pathway [21]. In a Review by Plourde and Cunnane [24] the authors discuss the acceptance of the view that there is an “extremely limited efficiency” of the desaturase conversion of ALA to DHA. The controversy of the conversion of LA and ALA to the long chain PUFAs arose in part due to the early use of rat models and also due to using animals that were deficient in essential fatty acids [25]. These two approaches were misleading as rats have a more efficient conversion of LA and ALA to longer chain PUFA, and fatty acid deficiency stimulates the conversion of LA and ALA to longer chain PUFA [25]. Although EPA and DHA biosynthesis is generally regarded as being inefficient [22], the extent of this inefficiency is controversial as measurements of longer chain PUFA may be quite different in plasma *versus* levels measured in other tissues, and it is plasma levels that are more commonly measured and reported [24,26]. However, it seems that the most predictable means of achieving adequate levels of EPA and DHA in plasma and tissues is through consuming long chain PUFA from dietary sources.

The intake of animal, saturated and *trans*-unsaturated fats is associated with all-cause mortality [27] and death due to prostate cancer [18,27]. The consumption of MUFA, PUFA, and vegetable fats on the other hand are associated with a decreased risk of developing prostate cancer or death from prostate cancer [18,27].

Unrepaired DNA damage can result in mutations and some mutations can lead to the development of cancerous tumours. Polymorphisms, such as the single nucleotide polymorphism rs2853826 found in the mitochondrial gene *NADH dehydrogenase subunit*, can influence oxidative stress in women carrying the G allele (G10398), and who also consume alcohol [28]. In such an instance, genotype and alcohol consumption may therefore have an impact on the risk of breast cancer development. Unsurprisingly, cancers such as prostate cancer have been found to be associated with raised levels of DNA damage [29], and raised antioxidant levels can help activate the expression of the *glutathione S-transferase* gene and thereby help protect against this damage [30]. The measurement of DNA damage is regarded as an ideal biomarker for the assessment of the influence of foods or food components on cancer, and the alkaline comet assay (single cell gel electrophoresis) is regarded as a suitable technique for such an assessment [31,32].

The aim of this study was to determine the association between fat and oil intake, as part of a modified Mediterranean style dietary intervention study, and whole blood fatty acid profiles and their association with markers of inflammation and DNA damage in men with prostate cancer. It was hypothesised that the proposed diet would be associated with improvements in PSA, CRP, DNA damage and whole blood fatty acid levels. Evidence obtained could be used to support the prescribed diet as the basis for dietary guidelines that may benefit men with prostate cancer in the future.

## 2. Experimental Section

Ethical approval was obtained from the Northern B Health and Disability Ethics Committee, Auckland, New Zealand (Ethics number NTY/11/11/109) to perform this study. Study volunteers were selected from an existing cohort of men with prostate cancer based on their Gleason scores, such that those with a Gleason score of 6 (3 + 3) and 7 (3 + 4) were invited to participate in this dietary intervention. Neither a control group free from prostate cancer, nor a control group with prostate cancer and following a standard diet were included. The dietary intervention was explained in detail and a hardcopy of the guidelines and a lengthy compilation of recipes were provided [33]. From the point of view of fat intake, volunteers were asked to adhere to the following guidelines: to include 30–50 g of mixed, unsalted seeds and nuts daily; to include 15 mL or more of extra virgin olive oil and to avoid exposure of the oil to medium and high heat; to reduce dairy intake to one portion daily (information on alternative sources of dietary calcium was provided); to substitute butter and/or margarine with an olive oil based spread; to limit intake of red meat to less than 400 g a week and to substitute with oily fish and white meat; to avoid high temperature cooking of protein; to avoid processed meats; and to include oily fish in the diet at least once a week. The intention was not to change calorie intake, although there was a concern that this may increase due to nut and olive oil intake. Exercise was monitored at baseline and study end through the use of activity diaries. Light to moderate exercise was encouraged during the enrollment interview to encourage general well-being, but no support or resources were provided in this regard. Volunteers were provided with food samples due to the expense and novelty of some of the items, and blood samples were collected at baseline and at three months from volunteers in a non-fasting state. The blood samples were collected into vaccutainers and either kept on ice or at room temperature (plain, EDTA, Heparin and SST II Advance tubes were used). All blood tubes were processed within two hours of blood draw. The food samples supplied included 200 g of frozen vacuum packed salmon per week (Aoraki Smokehouse Salmon, Twizel, New Zealand) and 1 L of extra virgin olive oil (oleic acid content of 78.3%) per month (Seed Oil Extraction Ltd., Ashburton, New Zealand). Adherence to various aspects of the dietary intervention was assessed using a modified, validated questionnaire [34].

The fatty acid profiles were determined using the Holman Bloodspot fatty acid profile test (Lipid Technologies LLC (Austin, MN, USA) via Functional and Integrative Medicine Ltd. (Napier, New Zealand)). Frozen whole blood was thawed and approximately 75 μL was spotted onto the supplied filter cards. The composition of the fatty acids in the samples was derivatised to form fatty acid methyl esters and thereafter assessed using gas chromatography (Lipid Technologies LLC).

The comet assay can be used to detect lesions in DNA strands [35], and was used herein to assess change in DNA damage over time. Results were also obtained by additionally challenging DNA with hydrogen peroxide (H_2_O_2_) as described by Olive & Banáth [36]. This involved treating 20 μL of whole blood with 1 mL of a 200 μM solution of H_2_O_2_ in phosphate buffered saline solution, placing on ice for 30 min and discarding the supernatant after centrifugation. Thereafter the comet assay was performed on heparinised blood as outlined in Karunasinghe *et al.* [37,38]. DNA damage was quantitated using the Komet^®^ version 6.0 digital imaging system (Andor Technology, Belfast, UK). The first 50 leucocytes suitable for capturing were scored. Leucocytes were visualised using an Axioskop 2 fluorescent microscope (Zeiss, Goettingen, Germany) and a CCD camera (Evolution VF, QI Imaging, Media Cybernetics, Warrendale, PA, USA). In this way DNA damage was induced wherever significant weakness was present in the DNA strands and hence H_2_O_2_-induced DNA damage was considered as an indicator of “DNA fragility”. Data for percentage tail DNA were log-transformed as they were not normally distributed. The back-transformed mean of the log-transformed values was used for the statistical analysis.

Statistical analysis was carried out using SAS (V9.2 SAS Institute, Cary, NC, USA) as follows: the Students paired *t*-test was used for the comparison of variables at the baseline and three month time points and Spearman bivariate correlations were used to measure relationships between variables.

## 3. Results

The characteristics of the study participants are presented in Table 1 and summarised as follows: participants were aged between 52 and 74 years; 80% had a body mass index (BMI) of ≥25 kg/m^2^ and over the course of the study mean body weight reduced by 2.3 kg (*p* = 0.0007); 60% had undergone prostatectomy, whilst 30% of participants were on watchful wait or active surveillance. All participants had a Gleason score of 6 (3 + 3) or 7 (3 + 4) at the time of prostatectomy or most recent biopsy.

**Table 1:** Baseline characteristics of the study participants.

Baseline Characteristics		*n*
Age (years) (range 52–74 years)	50–59	3
	60–69	12
	≥70	5
BMI (kg/m^2^) (range 23–33 kg/m^2^)		
	≤19.9	0
	20–24.9	4
	25–29.9	12
	≥30	4
Gleason score *	3 + 3	14
	3 + 4	6
Smoking status	Never	7
	Past	13
	Present	0
Supplements	Omega 3 (from fish oil)	3
	Vitamins	4
Treatment type	None	6
	Prostatectomy	10
	Prostatectomy + ADT + DxR	1
	Prostatectomy + DxR	1
	ADT + DxR	1
	Brachytherapy	1

BMI: Body mass index; ADT: Androgen deprivation therapy; DxR: Radiotherapy (other than Brachytherapy); * The Gleason score is based on tissue obtained from the prostatectomy. Where a prostatectomy was not performed, the Gleason score was based on a biopsy sample.

**Figure 2:**
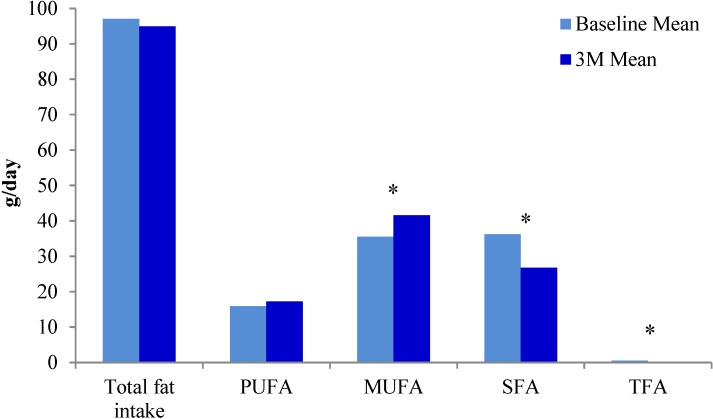
Changes in types of dietary fat intake from baseline to three months.* Statistically significant *p* values; PUFA: polyunsaturated fatty acids; MUFA: monounsaturated fatty acids; SFA: saturated fatty acids; TFA: trans fatty acids.

A modified Mediterranean adherence score was used to assess adherence to the study diet at baseline and at three months. The intake of olive oil, nuts, dairy, fish and red meat changed significantly over the course of the study (Table 2). Saturated fat intake, as a percentage of total fat intake at baseline and three months, decreased significantly (*p* < 0.0001). As expected, the source of dietary fat changed in response to the recommended dietary intervention. Figure 2 shows intake of MUFA increased and SFA and total fatty acid decreased significantly over the study period. However, the intake of total fat and PUFA, when measured in grams per day, did not change (Figure 2).

The source, type and amount of fatty acid intake influenced various physiological characteristics, as well as blood levels and ratios. At study end BMI was inversely and significantly correlated to blood *n*3PUFA (*r* = −0.451; *p* = 0.046). Decreases in BMI were associated with increased measurements of PUFA (*r* = −0.484; *p* = 0.031) and LA (*r* = −0.463; *p* = 0.040). In addition, increased whole blood arachidonic acid (AA) (*r* = −0.455; *p* = 0.044) levels were associated with weight loss but not a significant decrease in BMI.

**Table 2:** Changes in the sources of dietary fat from baseline to three months.

Dietary Component (Unit of Measure)	Mean (SE)		Mean Difference (95% CI)	*p*
	Baseline	Three Months		
Olive oil (mL/day)	14.5 (3.8)	28.8 (4.7)	14.2 (6.8–16.0)	**0.0008**
Nuts (Servings/week)	2.3 (0.5)	5.1 (0.6)	2.9 (1.5–4.2)	**0.0003**
Butter/cream/margarine (Servings/day)	2.1 (0.3)	1.0 (0.3)	−1.1 (−0.6–−1.6)	**0.0002**
Dairy products (Servings/week)	7.4 (0.9)	4.4 (0.7)	−2.9 (−1.2–−4.7)	**0.0025**
Fish (Servings/week)	1.7 (0.2)	3.5 (0.5)	1.8 (0.9–2.7)	**0.0005**
Red and processed meat (Servings/week)	3.9 (0.5)	1.9 (0.4)	−2.0 (−2.6–−1.3)	**0.0005**

SE: standard error; CI: confidence interval.

Total SFA significantly decreased at study end, partly due to a significant decrease in stearic acid intake (Table 3). Total MUFA, PUFA or any individual fatty acid within those synthesis pathways, showed no change, with the exception of DHA and DHA + EPA which showed a statistically significant increase in blood levels (Table 3). In addition, the ratios of *n*6PUFA:*n*3PUFA and AA:EPA had both decreased by study end (Table 3).

No significant correlations were noted between fatty acid measurements obtained from the blood fatty acid profile and food intake assessed via FoodWorks^®^7 (Xyris software Pty Ltd. 2012, Kenmore Hills, Australia). However, some statistically significant correlations were evident between various fatty acids reported from the blood fatty acid profile and food items as assessed in an adherence questionnaire (Table 4). Dairy intake in particular was inversely correlated with total *n*3PUFA, EPA and EPA + DHA, and positively correlated with the ratio of AA to EPA (Table 4).

C-reactive protein, PSA and DNA damage were measured at baseline and at three months. Neither C-reactive protein nor PSA changed significantly over the course of the study period. However, a significant, inverse relationship between adherence to the modified Mediterranean diet and basal DNA damage emerged. Spearman correlation was used to identify relationships between intake of individual food items that were recommended as part of the dietary intervention and DNA damage at three months. Foods high in animal fat were significantly positively associated with basal DNA damage (Table 5). In addition, association of DNA fragility with various fat related dietary components was assessed and the DNA fragility was inversely correlated with fish intake (*r* = −0.452; *p* = 0.045) whilst dairy intake was found to be positively associated with DNA fragility (*r* = 0.571; *p* = 0.008).

**Figure 3:**
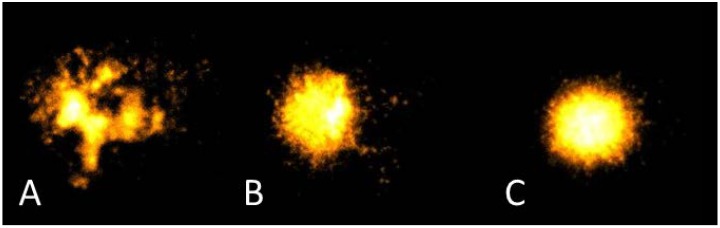
Representative images of different levels of DNA damage as measured by the Comet assay. **A**: extensive damage; **B**: moderate damage; **C**: minor damage.

Significant correlations were observed between basal DNA damage and dietary fat sources, as measured by an adherence questionnaire, as well as various fatty acids reported from the blood fatty acids profile at three months (Table 5). No associations were evident when analysing fatty acid intake, as measured by the diet diaries and analysed via FoodWorks^®^7 (Xyris software Pty Ltd. 2012), and basal DNA damage. A representative example of various levels of DNA damage is evident in Figure 3.

Results show that total MUFA and *n*9MUFA (particularly oleic acid), were inversely associated with DNA damage while total *n*6PUFA, and a higher ratio of *n*6PUFA to *n*3PUFA, were associated with increased DNA damage.

**Table 3:** Whole blood fatty acid profile expressed as mean percent, at baseline and three months.

Blood Fatty Acids	Mean (SE)		Mean Difference (95% CI)	*p*
	Baseline	Three Months		
Total SFA	34.7 (0.3)	33.7 (0.4)	−1.0 (0.4–1.5)	**0.002**
16:0 Palmitic acid	22.6 (0.3)	22.3 (0.4)	−0.3 (−0.1–0.7)	0.161
18:0 Stearic acid	10.5 (0.2)	10.0 (0.2)	−0.5 (0.2–0.9)	**0.002**
Total MUFA	23.4 (0.4)	23.7 (0.4)	0.3 (0.4–1.0)	0.366
Total *n*9MUFA	23.1 (0.4)	23.4 (0.4)	0.3 (−0.4–1.0)	0.380
18:1ω9 Oleic acid	22.7 (0.4)	23.2 (0.4)	0.5 (−0.2–1.1)	0.162
Total PUFA	39.5 (0.5)	40.3 (0.5)	0.9 (−0.1–1.8)	0.079
Total *n*6PUFA	32.8 (0.4)	33.0 (0.5)	0.2 (−0.7–1.2)	0.636
18:2ω6 LA	19.6 (0.7)	19.4 (0.9)	−0.2 (−1.7–1.4)	0.832
20:4ω6 AA	9.1 (0.3)	8.9 (0.3)	−0.2 (−0.7–0.3)	0.379
Total *n*3PUFA	6.6 (0.4)	7.3 (0.3)	0.6 (−0.0–1.3)	0.057
18:3ω3 LNA	0.5 (0.0)	0.6 (0.1)	0.0 (−0.1–0.2)	0.689
20:5ω3 EPA	1.4 (0.9)	1.5 (0.7)	0.1 (−0.2–0.5)	0.463
22:6ω3 DHA	3.0 (0.9)	3.5 (0.1)	0.5 (0.2–0.8)	**0.001**
EPA + DHA	4.4 (0.4)	5.0 (0.2)	0.6 (0.3–1.2)	**0.042**
Modified WBS *n*3 Index	6.1 (0.5)	7.0 (0.3)	0.9 (0.0–1.7)	**0.043**
*n*6PUFA:*n*3PUFA	5.2 (0.3)	4.7 (0.2)	−0.6 (−1.0–−0.1)	**0.019**
AA:EPA	8.58 (0.9)	6.9 (0.6)	−1.6 (−3.1–−0.2)	**0.030**

Abbreviations: AA: Arachidonic acid; CI: confidence interval; DHA: docosahexaneoic acid; DPA: docosapentaenoic acid; EPA: eicosapentaenoic acid; LA: linoleic acid; LNA: linolenic acid; MUFA: monounsaturated fatty acids; *n*9MUFA: omega 9 monounsaturated fatty acids; *n*3PUFA: omega 3 polyunsaturated fatty acids; *n*6PUFA: omega 6 polyunsaturated fatty acids; *p*: probability value; PUFA: polyunsaturated fatty acids; SE: standard error; SFA: saturated fatty acids; WBS: whole blood spot.

**Table 4:** Correlation between various whole blood fatty acid levels and intake of selected food items at three months.

Blood Fatty Acids	Dietary Fat Source	Correlation	*p*
Total *n*3PUFA	Fish intake	0.210	0.374
	Nut intake	0.341	0.141
	Dairy intake	−0.433	0.057
	Red meat intake	0.082	0.732
EPA	Fish intake	0.172	0.468
	Nut intake	0.147	0.535
	Dairy intake	−0.580	**0.007**
	Red meat intake	−0.475	**0.034**
EPA + DHA	Fish intake	0.123	0.605
	Nut intake	0.222	0.347
	Dairy intake	−0.609	**0.004**
	Red meat intake	0.055	0.817
*n*6PUFA:*n*3PUFA	Fish intake	0.192	0.418
	Nut intake	−0.349	0.132
	Dairy intake	−0.147	0.537
	Red meat intake	0.486	**0.029**
AA:EPA	Fish intake	0.233	0.323
	Nut intake	0.084	0.725
	Dairy intake	0.409	0.073
	Red meat intake	−0.029	0.904

Abbreviations: EPA: eicosapentanoiec acid; DHA: docosahexaneoic; *n*3PUFA: omega 3 polyunsaturated fatty acid, *n*6PUFA: omega 6 polyunsaturated fatty acid; AA: arachidonic acid.

**Table 5:** Correlation between DNA damage and dietary fatty acid intake and blood fatty acids.

Outcome of Interest	Dietary Fat Sources	Baseline		Three Months	
		Correlation	*p*	Correlation	*p*
DNA damage	Olive oil	0.002	0.995	−0.370	0.109
	Servings of butter, cream, margarine	0.278	0.235	0.456	**0.043**
	Servings of fish	0.202	0.393	0.510	0.829
	Servings of red meat	0.066	0.783	0.576	**0.007**
Blood Fatty Acid	Total MUFA	0.200	0.3988	−0.565	**0.009**
	Total *n*9MUFA	0.211	0.371	−0.561	**0.010**
	Oleic acid	0.220	0.352	−0.514	**0.020**
	Total *n*6PUFA	−0.116	0.627	0.536	**0.015**
	Total *n*3PUFA	−0.314	0.178	−0.224	0.342
	*n*6PUFA:*n*3PUFA ratio	0.330	0.155	0.507	**0.023**

Abbreviations: MUFA: mono-unsaturated fatty acids; *n*9MUFA: omega 9 polyunsaturated fatty acids; *n*6PUFA: omega 6 polyunsaturated fatty acids; *n*3PUFA: omega 3 polyunsaturated fatty acids.

## 4. Discussion

Dietary fat intake was measured by assessing whole blood fatty acid levels, as well as by using four-day food diaries and assessing intake via FoodWorks^®^7 software (Xyris software Pty Ltd. 2012). A modified Mediterranean diet adherence questionnaire was used to evaluate conformity to a Mediterranean style dietary pattern, which is generally high in both *n*3 and *n*6PUFA, and to measure intake and change in intake of specific high fat foods in response to the dietary intervention.

The Holman Bloodspot fatty acid profile test (Lipid Technologies LLC), requiring whole blood samples, was used to assess fatty acid profiles at baseline and study end. Although other fatty acid profile tests can be used to measure fatty acid levels from other components of blood samples, whole blood was regarded as preferable as it can be used to assess fatty acid intake over the previous two months. Both Rise *et al.* [39] and Sun *et al.* [40] state that erythrocyte fatty acid profiles provide a better reflection of long term PUFA intake than plasma fatty acid profiles, and this view is supported by work carried out by Katan *et al.* [41]. In their study, Katan *et al.* concluded that erythrocyte fatty acid profiles reflected intake over the past one to two months [41]. It is clear that plasma and serum fatty acid profiles reflect more recent fatty acid intake than erythrocyte, whole blood or adipose tissue fatty acid profiles [39,40,42]. Based on this evidence it is likely that the Holman Bloodspot test captures fatty acid intake over the two months prior to blood collection.

A number of statistically significant changes in fatty acid profiles (Table 3) were noted such as the increase in DHA whole blood levels from 3.0% to 3.5% (*p* = 0.001). However, although the changes in EPA levels were not statistically significant, they did increase from 1.36% to 1.5% over the three month study period. Together, these changes contributed to a statistically significant increase in the modified WBS *n*3 index (*p* = 0.043) (Table 3).

Fatty acid profiles were measured from whole blood spots (WBS) and this presents challenges with regards to calculating a red blood cell (RBC) *n*3 index. A RBC *n*3 index is typically calculated from the sum of EPA and DHA (from RBC membranes) as a percentage of total RBC fatty acids [43]. Bailey-Hall *et al.* compared DHA and EPA levels from whole blood obtained from a finger prick with values obtained from RBCs (venipuncture) [44]. Although the mean percentage for DHA was approximately 150% lower from capillary whole blood than in RBCs, the DHA and EPA values from the two sample types were highly correlated [44]. As mentioned in the “Experimental” section, the fatty acid profiles were determined by Lipid Technologies LLC (Austin Minnesota) using Holman Bloodspot fatty acid profile tests. Results were presented as a percentage of total lipid content from WBS. An RBC *n*3 index was reported and as this percentage was calculated from WBS, it was therefore necessary to apply a conversion factor wherein the relationship between DHA from whole blood *versus* DHA from red blood cells, was taken into account [44]. The actual algorithm used is proprietary information [45]. Rather than refer to this value as the RBC *n*3 index, which could be viewed as misleading, the authors have used the term “modified WBS *n*3 index” (Table 3). Due to the relationship between blood fatty acids in whole blood *vs.* RBC from venipuncture established by Bailey-Hall [44] upon which the algorithm developed by Lipid Technologies is based [45], the authors have considered the modified WBS *n*3 index as equivalent to the widely used RBC *n*3 index. The RBC *n*3 index is negatively associated with death, particularly sudden death, from coronary heart disease [43]. Although the most desirable levels might be influenced by cultural background, maximal cardioprotection and slowest rate of telomere loss takes place at an RBC *n*3 index ≥8% and 8.7% respectively [43]. The increase in the modified WBS *n*3 index found in this study (from 6.10% to 6.98%) was significant, yet it remained below the target value for the reduction of coronary heart disease risk. However, it is believed that any increase in the modified WBS *n*3 index would be beneficial as *n*3 fatty acids can alter membrane biophysical properties and in addition to lipid metabolism, this may impact on inflammatory responses [46]. In addition, the dietary intervention continued for only three months and it is possible that the modified WBS *n*3 index may have continued to increase until target levels were reached.

Fatty acid profiles have predominantly been analysed from either plasma [47,48,49] or serum [50] and therefore results from these studies are not comparable with our own due to the different substrates used. However, in a recent Australian study data were collected on fasting whole blood fatty acids, but only intake in grams of SFA, MUFA and PUFA were shown, as well as a limited number of blood fatty acid ratios [51]. Total fat, as well as SFA, MUFA and PUFA intake (all measured in grams per day) were all comparatively higher in our study, relative to the study carried out by Alhazmi *et al.* [51].

An association between a change in dietary pattern over a three month time period, and whole blood fatty acids was investigated. Importantly, overall fat intake did not change despite a Mediterranean dietary pattern being traditionally high in fat. This lack of change is due to a substitution in source of fats, such that meats high in saturated fat were replaced by oily fish, and although dairy intake decreased significantly, olive oil and nut intake also increased significantly (Table 2). These changes are consistent with the adoption of a Mediterranean style dietary pattern. The change in fat source is supported by the statistically significant decrease in total SFA (Figure 2), particularly stearic acid (Table 3). Changes in blood fatty acid profiles, although physiologically small, were statistically significant (Table 3). This is largely due to the fact that the percentage values are small and therefore a large physiological change is unlikely.

In this dietary intervention study the intake of olive oil, oily fish, seeds and nuts was promoted, and therefore the dietary intake of MUFA and PUFA increased (Table 2). This increase is partly reflected in the change in blood fatty acid levels (Table 3). The intake of dietary sources of MUFA increased significantly (*p* = 0.0243) (Figure 2), as did the whole blood levels of *n*3PUFAs DHA (*p* = 0.001) and EPA + DHA (*p* = 0.042) (Table 3).

The increase in the modified WBS *n*3 index was consistent with the reported intake of dietary items containing *n*3PUFAs. Increased intake of *n*3PUFA is often associated with a reduction in *n*6PUFA blood levels partially due to competitive inhibition of rate limiting desaturase enzymes [23] (Figure 1), although there is some debate regarding this perhaps overly simplistic view [24,26]. However, there were no correlating significant changes in percentage *n*6PUFA in our study. While we expected that intake of some sources of *n*6PUFA, such as the cheaper vegetable oils that are often found in processed foods (e.g., soybean, sunflower, rice-bran, cottonseed and corn oils) would decrease due to substitution with olive oil, which is much lower in *n*6PUFA, there is no evidence that this occurred. Although we assessed for olive oil intake, we did not question the intake of other oils. An alternative explanation could lie in the fatty acid composition of nuts. We recommended and observed an increased consumption of nuts. Nut consumption increased from a mean of 2.2 to 5.2 servings per week. Many nuts are high in *n*6PUFA, thus off-setting the decrease of *n*6PUFA from other sources. In spite of this, the increase in *n*3PUFA contributed to a statistically significant decrease in the *n*6:*n*3 ratio, indicating a shift towards a less inflammatory profile. Correlations between intake of dietary fatty acids and blood fatty acids were not evident (values not reported). The levels of blood fatty acids are not only affected by intake [48,49], but also by the rate at which fatty acids are transformed (Figure 1). This transformation is often inefficient and influenced by rate limiting enzymes such as the delta-6-desaturase enzymes [52].

It is also important to consider whether *n3*PUFAs from plant sources decreased as this might counter-balance the increase in fish intake. However, one would expect this to be evident from the blood fatty acid profiles, as sources of EPA and DHA would largely be from oily fish and the limited conversion of alpha linolenic acid via elongation and desaturation reactions to stearidonic acid, EPA and finally DHA [23]. EPA and DHA can also be obtained from certain algal species [23], but only one of the study participants took algae-based supplements. The output from FoodWorks^®^7 software (Xyris software Pty Ltd. 2012) is in the form of food components and therefore we are comparing measurements of whole foods, such as fish, from the adherence tool, with measurement of food components such as *n*3PUFA, which can be sourced from a number of different foods including fish, refined vegetable oils and nuts for example.

What is of particular interest and relevance is that EPA intake was inversely associated with intake of dairy products (*p* = 0.007) and red meat (*p* = 0.034); and blood percentage EPA + DHA was significantly inversely associated with dairy intake. Due to the study design, the effect of dietary intake on prostate cancer risk could not be assessed. However, it is interesting to note that the above mentioned association between increased EPA and DHA intake with decreased dairy was also reported in a study where the influence of various dietary components on prostate cancer risk was assessed [53]. These results support evidence obtained from the adherence questionnaires that fish, as the primary dietary source of EPA, partially replaced the intake of meat, and some dairy products.

The Comet assay is a standard method for measuring DNA damage in eukaryotic cells, regardless of how that damage has been caused [54]. Leucocytes, as in this study, are usually used for the analysis of comets, but one of the drawbacks is that these cells are not usually a target tissue for cancer [54]. However, DNA damage in leucocytes, as measured in a Comet assay, may still present as a reliable marker for increased cancer risk as genomic instability is a common and widely accepted characteristic amongst cancers. A number of studies have been reported wherein DNA damage has been used to assess response to genotoxic stress in terms of cancer risk or effect on cancer related pathways [55,56,57]. Machowetz *et al.* and Colomer *et al.* both reported a reduction in DNA damage in response to olive oil consumption [58,59]. For this reason it was anticipated that a similar reduction in DNA damage would be observed in our own study participants as their consumption of extra virgin olive oil had increased significantly from 14.83 mL/day to 28.75 mL/day (Table 2). While an inverse association was seen between olive oil consumption and DNA damage, this was not significant (*p* = 0.109) (Table 5). However, the percentage of oleic acid in the blood (along with total MUFA and total omega 9), was inversely associated with basal DNA damage at the end of the study, which is consistent with published results [60]. As this association occurred in spite of only a minor increase in the blood oleic acid ratio, the relationship may serve as a marker for an unmeasured, associated factor, such as olive oil polyphenols.

When investigating food sources of fatty acids, it was clear that DNA damage was associated with a higher intake of dairy products and red meat (Table 5). Increased MUFA intake (Figure 2), supported by statistically significant MUFA blood levels (Table 3) were inversely correlated with basal DNA damage at three months (Table 5). Total *n*6PUFA and *n*6PUFA:*n*3PUFA on the other hand were positively correlated with DNA damage (Table 5) and this was not unexpected as *n*6PUFA is believed to be pro-inflammatory and low *n*6PUFA:*n*3PUFA ratios are believed to be anti-inflammatory.

While we did not question participants to obtain detailed information about culinary fats at baseline, we predicted that most participants would have been consuming olive oil with a lower level of polyphenols than that provided by the extra virgin olive oil supplied for this study (Oil Seed Extractions Ltd., Ashburton, New Zealand). Furthermore, our requirement of just one or more tablespoons of olive oil daily was perhaps too low to boost oleic acid levels sufficiently. In a study by Mitjavila *et al.* [61] olive oil was supplemented at a rate of a litre per week (equivalent to just over 140 mL/day), the usual Mediterranean diet includes 60 mL/day of extra virgin olive oil [34]. However, this was thought to be too high an expectation for a New Zealand population that does not have a tradition of olive oil consumption.

The inclusion of oily fish was an important component of the modified Mediterranean diet. The diet was modified to promote the inclusion of oily fish due to the *n*3PUFA content in fish being a good source of the anti-inflammatory fatty acids, EPA and DHA. From the adherence questionnaire the reported intake of fish doubled (Table 2), and this increase was statistically significant (*p* = 0.0005). No significant correlation was seen between any of the blood fatty acids and fish intake (correlations ranged from *r* = 0.017 to 0.21 (Table 4)). These results are not entirely inconsistent with those reported by Norrish *et al.*, in which fish intake was “moderately correlated” to EPA and DHA when measured from red blood cells obtained from New Zealand men (*r* = 0.26 and 0.32 respectively, the *p* values were not reported) [62]. The whole blood fatty acid profile is a reflection of oily fish intake over the preceding two months, whilst the diet diaries are a measure of intake the week prior to the blood draw. Some of the volunteers indicated that they had consumed all their salmon donations by this stage and may have been unwilling to purchase additional oily fish. This highlights the advantage of blood biomarkers that reflect both short and longer-term intake over diet diaries or food frequency questionnaires to assess dietary intake.

In addition to changes in the consumption of fish, it can be seen that intake from other sources of dietary fat also changed. Statistically significant changes were seen in the consumption of olive oil and nuts, where consumption increased, and dairy products, where consumption decreased (Table 2). This could result in the increased intake of *n*3PUFA and the decreased intake of *n*6PUFA, depending on the type and quantity of nuts consumed. The type of nuts consumed was not recorded.

Although the authors cannot speculate as to whether the modified Mediterranean diet detailed herein would increase longevity, it is clear that indicators of general health were enhanced. This view is supported by the fact that many of the men who were carrying excess weight, decreased their body weight during the study period; that whole blood fatty acid profiles improved, specifically DHA levels and the modified WBS *n*3 index (a marker of heart health); and that DNA damage levels decreased. In addition, anecdotal reports show that one of the study volunteers reported improved sleep patterns, thought to be due to decreased nocturia (nocturia being a common side-effect of prostate cancer treatment and prostatic disease); one volunteer experienced reduced arthritic pain; another experienced a reduced need for anti-inflammatory medication, whilst a number of volunteers commented on an improved feeling of well-being.

## 5. Conclusions

Dietary change to promote the intake of oily fish and olive oil as part of a Mediterranean style diet can be achieved in men with prostate cancer. Both the source and type of dietary fat intake changed significantly over the course of the dietary intervention. The intake of olive oil, nuts and fish increased significantly, whilst the intake of dairy and red meat decreased significantly from baseline to three months. The whole blood levels of the SFA, stearic acid decreased significantly, whilst the levels of DHA increased significantly. Although the whole blood levels of total *n*6PUFAs did not change significantly over the course of the intervention, care should be taken to provide advice regarding the increased intake of nuts to ensure that the type and quantity of nuts consumed maintains *n*6PUFA within levels associated with reduced health risks. Whilst dietary fat intake significantly changed over the course of the study, this change was not statistically associated with the significant changes in blood fatty acid profiles. However, total MUFA and oleic acid levels in the volunteers adhering to this dietary intervention were associated with a significant reduction in DNA damage. DNA damage was positively correlated with the ratio of *n*6PUFA to *n*3PUFA, as well as to the intake of red and processed meats, and dairy products.
